# Patient-Reported Outcomes as Endpoints in Clinical Trials of Kidney Transplantation Interventions

**DOI:** 10.3389/ti.2022.10134

**Published:** 2022-05-20

**Authors:** Allison Tong, Rainer Oberbauer, Maria Irene Bellini, Klemens Budde, Fergus J. Caskey, Fabienne Dobbels, Liset Pengel, Lionel Rostaing, Stefan Schneeberger, Maarten Naesens

**Affiliations:** ^1^ Sydney School of Public Health, The University of Sydney, Sydney, NSW, Australia; ^2^ Department of Nephrology and Dialysis, Medical University of Vienna, Vienna, Austria; ^3^ Department of Surgical Sciences, Sapienza University of Rome, Rome, Italy; ^4^ Department of Nephrology and Medical Intensive Care, Charité Universitätsmedizin Berlin, Berlin, Germany; ^5^ Department of Population Health Sciences, University of Bristol, Bristol, United Kingdom; ^6^ Department of Public Health and Primary Care, Academic Center for Nursing and Midwifery, KU Leuven, Leuven, Belgium; ^7^ Centre for Evidence in Transplantation, Nuffield Department of Surgical Sciences, University of Oxford, Oxford, United Kingdom; ^8^ Department of Nephrology, Dialysis and Transplantation, Toulouse University Hospital, Toulouse, France; ^9^ Department of General, Transplant and Thoracic Surgery, Medical University of Innsbruck, Innsbruck, Austria; ^10^ Department of Microbiology, Immunology and Transplantation, KU Leuven, Leuven, Belgium

**Keywords:** patient-reported outcome measure (PROM), patient perspective, adherence, life participation, SONG-Tx, PROMIS®

## Abstract

Patient-reported outcomes (PROs) that assess individuals’ perceptions of life participation, medication adherence, disease symptoms, and therapy side effects are extremely relevant in the context of kidney transplantation. All PROs are potentially suitable as primary or secondary endpoints in interventional trials that aim to improve outcomes for transplant recipients. Using PRO measures (PROMs) in clinical trials facilitates assessment of the patient’s perspective of their health, but few measures have been developed and evaluated in kidney transplant recipients; robust methodologies, which use validated instruments and established frameworks for reporting, are essential. Establishing a core PROM for life participation in kidney transplant recipients is a critically important need, which is being developed and validated by the Standardized Outcomes in Nephrology (SONG)-Tx Initiative. Measures involving electronic medication packaging and smart technologies are gaining traction for monitoring adherence, and could provide more robust information than questionnaires, interviews, and scales. This article summarizes information on PROs and PROMs that was included in a Broad Scientific Advice request on clinical trial design and endpoints in kidney transplantation. This request was submitted to the European Medicines Agency (EMA) by the European Society for Organ Transplantation in 2016. Following modifications, the EMA provided its recommendations in late 2020.

## Introduction

The importance of the patient’s perspective on their own health in the assessment of benefits and risks of therapeutic interventions is widely acknowledged (1). Such information could be relevant for drawing regulatory conclusions regarding treatment effects, benefit/risk balance assessments, or specific therapeutic claims (2). A patient-reported outcome (PRO) describes information assessed and reported directly by the individual about how they feel or function in relation to their health or treatment, without interpretation or modification by anyone else, including clinicians and researchers (1, 3). Examples of PROs include health-related quality of life (HRQoL), physical function, ability to work, specific symptoms related to the disease or its treatment (e.g., pain, fatigue, side effects), and treatment adherence. A PRO measure (PROM) is a standardized quantitative assessment that captures the impact of disease and treatment as perceived by the individual.

In clinical research, PROs may be used as primary, co-primary, secondary, or exploratory endpoints (1, 4). However, evidence for the psychometric robustness of PROMs is an important consideration for the selection of PROs as endpoints in trials. The European Medicines Association (EMA) guideline on clinical investigation of medicinal products for the treatment of rheumatoid arthritis, for instance, recommends that several PROs are considered for secondary or supportive endpoints (5). However, for most disease areas, PROs are rarely incorporated in drug labeling claims. For example, of 60 PRO claims in orphan drug applications approved by the EMA between 2012 and 2016, only 12 (21.7%) of the products contained PROs in clinical study sections of the Summary of Product Characteristics (SmPC) (6). In 12 SmPCs, PROMs were based on symptoms; five also utilized patient functioning. HRQoL-related claims were included in eight approvals. A PRO was the primary endpoint in SmPCs in four (31%), a secondary endpoint in eight (62%), and a tertiary endpoint in one of the 13 approvals with a PRO claim. PROs that were primary endpoints assessed disease-specific symptoms exclusively (6).

Likewise, PROs are infrequently reported in kidney transplantation trials. Although regulatory agencies increasingly support the inclusion of PROs in clinical trials, few studies of medication regimens in kidney transplantation conform to these recommendations. One systematic review, for example, reported that only 2% of maintenance immunosuppression studies in kidney transplantation reported HRQoL outcomes (7). Another systematic review of 397 trials involving 63,514 adult kidney transplant recipients found substantial variability in PROs being assessed, as well as in PROMs used; the most frequent PROs were pain (40 trials, 15 measures), adherence (15 trials, eight measures), sleep (11 trials, four measures), and fatigue (11 trials, five measures) (8). Heterogeneity in choice of PROMs makes it difficult to compare intervention effects across trials. The PRO Rosetta Stone project developed and applied methods to link the patient-reported outcomes measurement information system (PROMIS) with other relevant measures, to provide equivalent scores for different scales that measure the same outcome (9). Also, there is limited evidence on the psychometric properties of PROMs used in kidney transplant recipients (10, 11).

This article provides an evidence-based and recipient-centered overview of PROs to be included as primary, secondary, or exploratory endpoints in clinical trials of kidney transplantation. Guidance on PRO measurement is also included, and the need for reliable measurement of medication adherence in randomized controlled trials (RCTs) is discussed.

## PROs to Be Included in RCTs Involving Kidney Transplant Recipients

Two sections of the EMA’s CHMP guideline on clinical investigation of immunosuppressants for solid organ transplantation (11) refer briefly to the incorporation of PROs in RCTs. Section 4.3.2 (definition of secondary endpoints) mentions HRQoL in the list of other frequently reported endpoints that can be included, yet does not consider HRQoL as a mandatory primary or secondary outcome of RCTs within transplant recipient populations. Section 4.4.3b (therapeutic studies; confirmatory trials) mentions adherence in the first aim of product development based on comparative trials, namely, “to substitute one or several therapeutic components of well-established immunosuppressive regimens to improve efficacy, safety or compliance” (12).

Selecting the right PRO involves identifying outcomes that are important to individuals, in addition to what might be relevant to the study hypothesis and intervention. Based on consensus from transplant recipients, caregivers, and healthcare professionals (HCPs), the following PROs could be considered to be incorporated in RCTs of kidney transplantation interventions as primary or secondary outcomes: life participation; medication adherence; symptoms and side effects.

### Life Participation

**FIGURE 1:**
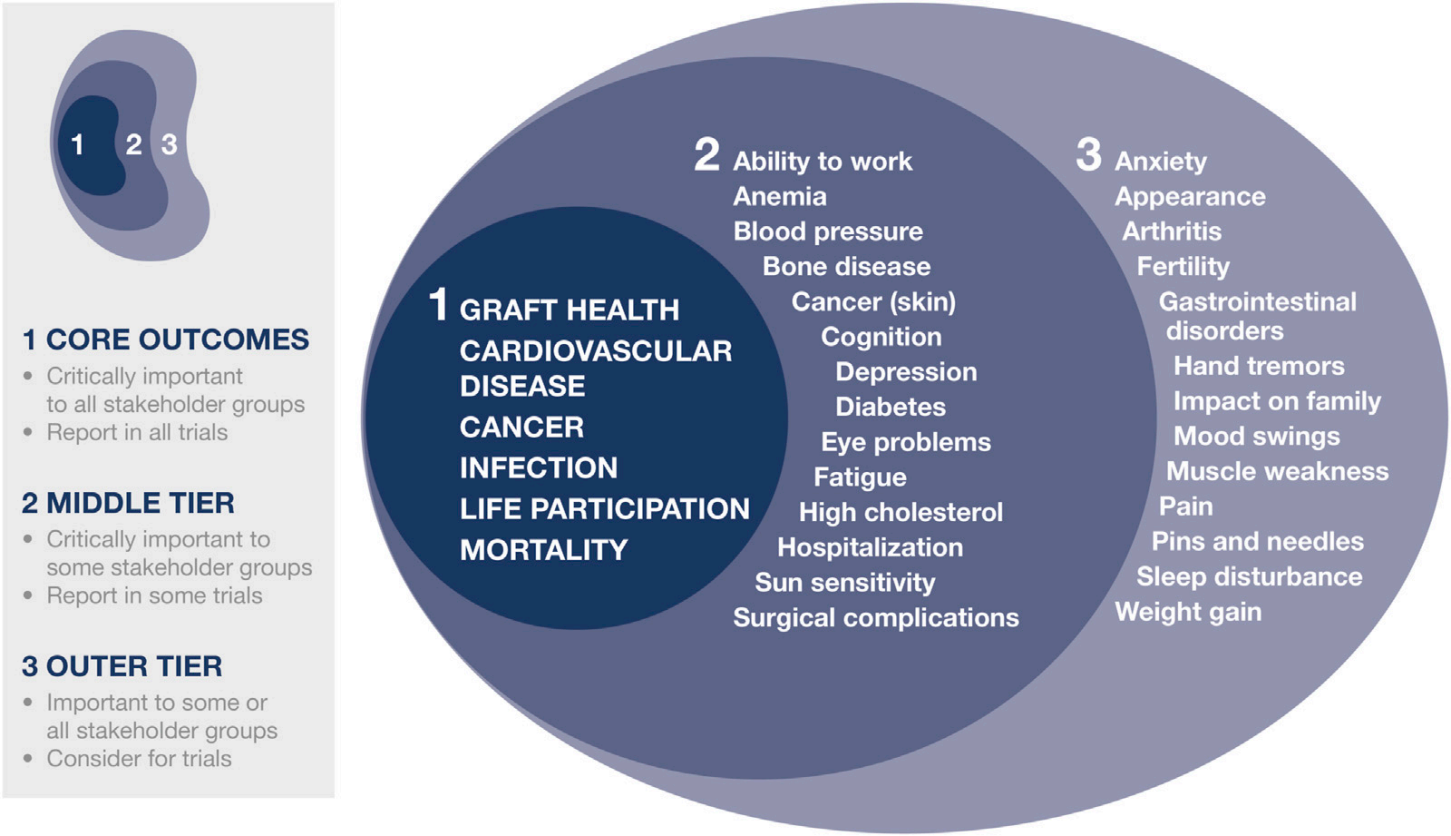
SONG-Tx core outcomes. Reprinted from Kidney Int. Vol 94. Tong A, et al. (13). Implementing core outcomes in kidney disease. Report of the Standardized Outcomes in Nephrology, with permission from Elsevier.

Through a consensus process involving over 1100 recipients, caregivers, and HCPs from 79 countries, the global Standardized Outcomes in Nephrology (SONG)-Tx initiative has established six core outcomes that should be reported in all kidney transplantation trials (Figure 1) (7, 8, 13). Alongside clinical outcomes relating to allograft loss, cardiovascular disease and mortality, cancer, and infection, life participation was the PRO of greatest importance to recipients, caregivers, and HCPs. Life participation describes “the ability to participate in activities that give patients a sense of fulfillment, enjoyment, control and hope in their lives” (14). Patients prefer not to specify life activities as these differ among individuals, so using a generic term enables life participation to be interpreted based on their own context (14).

### Medication Adherence

Non-adherence to prescribed medication is a global health concern. Adherence is divided into three quantifiable phases (15):

• Initiation (whether a patient takes the first prescribed dose)
• Implementation (the extent to which a patient’s actual dosing corresponds to the prescribed regimen, from initiation until last dose taken)
• Discontinuation (when no more doses are taken, with persistence indicating length of time between initiation and last dose taken).

#### Concerns Associated With Non-adherence

Annually, across Europe, medication non-adherence contributes to ∼200,000 avoidable deaths, and costs around €125 billion for excess hospitalizations, emergency care, and outpatient visits (16). Because of its impact on people’s health, the EU advocates improving medication adherence as a key policy lever to minimize waste and optimize value derived from pharmaceutical expenditure (17).

Medication non-adherence is a major concern in solid organ transplantation (18). To maintain allograft function, recipients are prescribed complex regimens, typically including immunosuppressants and drugs to prevent or treat comorbidities. On average, following kidney transplantation, recipients take 22 pills daily (range, 8–47) at 3 months, and 23 pills daily (range, 9–57) at 12 months (19), with ∼30% of the pill burden attributed to immunosuppressants (19, 20).

Compared with other solid organ transplant groups, kidney transplant recipients are the most vulnerable to non-adherence, with implementation problems occurring far more frequently than treatment discontinuation. Annually, over one-third of transplant recipients struggle to implement immuno-suppressive regimens correctly (21); deviations are commonly missed doses, incorrect dosing, or suboptimal timing of intake (22). Evidence consistently shows that poor implementation of an immunosuppressive regimen is an independent risk factor for rejection and allograft loss (23, 24). In addition, minor deviations from the regimen increase the risk of poor clinical outcome because of the narrow therapeutic window that exists for many immunosuppressant drugs (25).

The FDA supports the collection, analysis, and integration of patient perspective in the development of medical products and devices (26, 27). As part of their patient-focused drug development initiative, the US Food and Drug Administration (FDA) met with solid organ transplant recipients, caregivers, and advocates to elicit perceptions relating to recipients’ well-being and treatment (2). Participants deemed medication adherence to be important, yet strict regimens posed challenges because of the frequency and high quantities of drugs, the need for clinic visits to monitor allograft function, the impact of therapy side effects, and difficulty remembering to take medications. Participants expressed a need for therapies that maintain long-term organ function, have fewer long-term comorbidities (such as cancer), have fewer side effects, and offer reduced frequency of administration compared with standard of care (28). Besides simplifying regimens and reducing symptom burden, patients wanted individualized treatment.

Another FDA-convened open public workshop on antibody-mediated rejection in kidney transplantation, which involved participants from academia and industry in addition to transplant recipients, also concluded that the prevalence of non-adherence is high and must be addressed, to improve transplant outcomes (29).

#### Problems Associated With Assessing Adherence

When testing competing modes of drug treatment, it is essential to know the level of adherence to the regimen, including timely initiation, and punctual and sustained implementation, throughout the study.

Most deviations from a prescribed regimen can remain unnoticed yet jeopardize efficacy, safety, and selection of optimal dosing (15, 30–33). The gap between prescribed and actual drug-dosing history increases the risk of type II errors, as the combined effects of variable underdosing and increased variance in response weaken statistical power for any demonstration of efficacy (34).

Non-adherence might also result in higher doses being prescribed, to achieve target trough levels, which could increase the risk of toxicity in adherent patients (35).

Regulatory agencies acknowledge the importance of assessing adherence. In its industry guidance on RCTs to support drug approval and biological products for human use, the FDA recommends identifying and selecting transplantation candidates who are likely to adhere to the regimen, and advocates quantification of adherence throughout a study (36). Regulation 536/2014 of the European Parliament and the Council on Clinical Trials on Medicinal Products for Human Use also stipulates that the initial application dossier should include “a description of procedures for monitoring subject compliance, if applicable” (37). In Europe, the EMA also published the ICH E9 (R1) addendum on estimands and sensitivity analysis in RCTs to the guideline on statistical principles for clinical trials, requesting researchers to consider adherence when quantifying treatment effects (38).

Unfortunately, despite regulatory guidance, adherence is rarely given prominence in RCTs. Suboptimal measures continue to be used, as regulatory agencies provide no or limited guidelines on how adherence should be assessed. Although SPIRIT guidelines describe strategies to be applied within RCTs (to improve adherence to intervention protocols, and procedures for monitoring adherence) (39), SPIRIT is vague on how adherence is best assessed, and only provides examples of suboptimal measures (e.g., tablet return). We advocate reliable, quantifiable methods for adherence measurement in kidney transplantation later in this article.

### Patient-Reported Symptoms and Side Effects

The SONG-Tx initiative (13, 40) and the FDA meeting on patient-focused drug development and adherence (28) revealed that transplant recipients are concerned about the number and burden of side effects associated with immunosuppression. These include the onset of serious comorbidities and debilitating symptoms, such as fatigue or pain (Figure 1).

In RCTs, side effects are typically assessed by adverse event checklists, completed by the treating physician. Although adverse event reporting is vitally important to monitor safety, empirical evidence indicated that adverse event checklists identified only 7% of symptoms experienced by patients (30). A systematic review of adverse event reporting in 233 trials of maintenance immunosuppression following kidney transplantation found inadequacies including selective reporting, poor definition and description of measurement, and lack of alignment with known and common side effects (41). Consequently, in transplantation studies, the true burden of immunosuppressive regimens remains underestimated, in terms of the number and severity of adverse events, and the overall distress associated with treatment-related symptoms. Individuals may find it difficult to determine whether their symptoms relate to medications or their health condition (28), but irrespective of underlying causes, side effects and symptoms are important determinants of HRQoL, and might trigger non-adherence (42). PROMs can support patients in expressing how they feel and function so that, in turn, clinicians can aim to better manage patients’ symptoms (and how these impact on life), to improve patient-centered care. Therefore, we recommend that patient-reported symptoms and side effects represents meaningful primary or secondary endpoints for RCTs in kidney transplantation.

## Selection of Appropriate PROMs

Frameworks are available to guide the selection of PROMs for use in RCTs (3, 43–45). The rationale for selecting PROMs for a RCT should consider the prevalence and nature of the condition, characteristics that are relevant or unique to the patient population, patient perspectives and priorities, and outcomes that might be expected to change in response to the intervention (3, 45, 46).

PROMs can be classified into one of three categories (44). Firstly, there are generic health status measures, which assess a range of constructs [usually a combination of impairment, disability, and HRQoL (46)]. These can apply across different conditions or populations, are useful for broad comparisons of the relative impact of interventions between diseases, and can be compared with population normative data. Such measures include the 36-item Short Form Health Survey (SF-36), the World Health Organization Quality of Life Scale (WHO-QOL), and PROMIS®-29 (47). PROMIS-29 (and PROMIS-57) profile instruments that include the ability to participate in social roles and activities scale, and both have been validated in kidney transplant recipients (48, 49). Secondly, condition- or symptom-specific measures assess PROs within either a condition or disease, or across certain symptoms. Examples include the Kidney Disease Quality of Life instrument (KDQoL) (50), Kidney Transplant Questionnaire (51), Modified Transplant Symptom Occurrence and Symptom Distress Scale (52), End-stage Renal Disease Symptom Checklist—Transplantation Module (ESRD-SCLTM) (53), and Gastrointestinal Symptom Rating Scale (GSRS) (54). Finally, preference-based (or utility) measures assess a value (i.e., from <0 [worse than being dead] to 1 [full health]), assigned to the health state described by the patient. Values are assessed using direct methods (such as time trade-off or standard gamble), or multi-attribute utility instruments (55). A utility value allows comparison of HRQoL across conditions and between populations. In economic evaluations, these measures can be used to calculate quality-adjusted life-years (QALYs) to provide cost-effectiveness findings. Examples of preference-based measures include the EQ-5D, Health Utilities Index, and time trade-off calculations. In economic evaluations, such measures can be used to calculate QALYs and in doing so provide cost-effectiveness findings. Examples of preference-based measures are EQ-5D, HUI, and time trade-off. Data from the KDQoL/SF-36 and PROMIS profile measures can also be used in economic evaluations.

The UK Health Technology Assessment Programme recommends eight criteria for PROM selection: appropri-ateness, reliability (internal consistency, reproducibility), validity (criterion and predictive validity, face and content validity, construct validity), responsiveness, precision, interpretability, acceptability, and feasibility (43). COMET guidelines can be used to develop a core outcome set (COS), defined as a minimum set of outcomes that should be reported in all studies within a specific condition or population (56); in addition, the COnsensus-based Standards for the selection of health Measurement INstruments (COSMIN) initiative provides specific recommendations for selecting the most appropriate measures of COS (57) (https://www.cosmin.nl/). Core PROMs have been identified in kidney disease (e.g., fatigue in people undergoing hemodialysis) (58) and for other health conditions. The COMET and OMERACT initiatives recommend that a core outcome set includes a PRO (59) and there are frameworks for selecting core outcome measures for PROs (56, 59). Of note, the FDA has released guidance for core PROs in clinical trials in oncology (60). Below, we suggest measures to assess core outcomes for RCTs in kidney transplantation.

## Life Participation as a Core Outcome Measure

Following a consensus workshop on establishing a core outcome measure for life participation, kidney transplant recipients, caregivers, and HCPs recommended that such a measure needs to achieve several milestones. Firstly, it should capture recipients’ goals to fulfill their roles and re-establish a normal lifestyle post-transplantation. It should also include the diverse activities of “life” as defined by recipients, capture life changes caused by treatment complications and side effects, and be validated and feasible to implement (14).

A systematic review of 230 trials and observational studies found that 29 measures have been used to assess life participation in kidney transplant recipients (61). The most frequently used were the SF-36, KDQoL, and EQ-5D, which capture aspects of life participation in one attribute, although few instruments specifically measured aspects of life participation. Validation data were available for only six measures, and no validation data were available for the subscale capturing life participation. Also, none of the instruments adequately addressed recipients’ perspectives and experiences of life participation (14, 61). Establishing a core PROM for life participation in kidney transplantation populations is therefore needed, to ensure consistent reporting of this critically important outcome.

The SONG-Tx initiative suggested that PROMIS SF v2.0, *Ability to participate in social roles and activities*, was the best available measure to capture transplant recipients’ perspectives, priorities, and experiences regarding life participation (62). PROMIS items are available in ∼30 languages and have been rigorously validated (63); exploratory factor analysis, confirmatory factor analysis, item response theory modeling, and evaluation of differential item functioning were also used to test items (64). Cross-sectional evidence supports the validity of PROMIS items, and the reliability and precision of generic symptoms and functional reports: findings for PROMIS are comparable with other well-validated and widely accepted measures (65, 66).

Evidence also supports the psychometric robustness of PROMIS SF v2.0: sufficient unidimensionality, local dependence, monotonicity, graded response model item fit, and differential item functioning for age, sex, education, region, ethnicity, and language were demonstrated in a Dutch population (N = 1002) (67). Reliability, and content and construct validity, have been shown for PROMIS instruments (including ability to participate in social roles) in people with rheumatoid arthritis and cancer, and in clinical care settings (68).

The PROMIS measure was adapted by SONG-Tx following cognitive interviews with kidney transplant recipients [N = 20]. These were conducted using a pre-testing framework based on cognitive and social psychology, which assessed aspects of respondents’ comprehension, retrieval, response, and judgment (69). Initial findings indicated that kidney transplant recipients preferred positive wording compared with the focus on “trouble” used in the original PROMIS measure. In addition, if life participation is a primary outcome, use of a long measure is recommended, to facilitate comprehensive assessment (62). Following this preliminary work, items were adapted based on extensive input from kidney transplant recipients, with modifications being reviewed before generating the final Song-Tx Life Participation measure. A validation study in kidney transplantation is in progress and will be completed before recommending its use as a core outcome measure. More information on the Song-Tx Life Participation measure is available from the authors on request.

Other measures of health status or instruments assessing variables that might influence life participation (e.g., depression, fatigue) may also be required to address the specific aims of a study and/or intervention. For example, RCTs commonly include an economic evaluation to demonstrate cost-effectiveness, usually based on the benefits of the intervention, measured as QALYs. In this case, a utility-based instrument such as EQ-5D would be required, but this should be in addition to—not in place of—measuring life participation. We recommend that selecting additional PROMs should be undertaken in accordance with COSMIN guidelines or its equivalent.

## Measures of Medication Adherence

The COMMIT clinician group recommends measurement of medication adherence as the “fifth vital sign” in transplantation studies (18). Choice of method depends on phase of adherence under investigation (initiation, implementation, discontinuation), context of use (RCT, routine care, or registry), study purpose (observational or interventional), reliability and richness of data sought, participants’ preferences, and usability of the measures (70).

A systematic review of studies involving various chronically ill patient populations identified 20 different self-report measures for capturing non-adherence (71). The Basel Assessment of Adherence to Immunosuppressive Medication Se (BAASIS®; http://baasis.nursing.unibas.ch/) (72) was recommended for assessing adherence to immunosuppressive drug regimens because it is short to perform and easy to score, focuses on the implementation phase, considers both taking and timing of intake, and has established reliability and validity (71, 72). COMMIT also recommends using the BAASIS®, alongside the Insulin Treatment Appraisal Scale and Simplified Medication Adherence Questionnaire (18). However, more research on the prognostic value of self-reporting is needed, as PROMs might underestimate medication non-adherence.

Unlike self-report measures, smart technology allows for continuous measurement of adherence behaviors, providing objective data on day-to-day variability and timing of taking medication. Identifying the timing of gaps in drug exposure is needed when aiming to develop a reliable efficacy and safety profile. Smart technology is also recommended by the FDA to improve adherence measurement in RCTs (36). There are three broad categories of methods to measure adherence by means of smart technology (35). Firstly, video-assisted or photographic documentation of drug intake (e.g., using a mobile phone app with face-recognition technology, capturing the patient taking medication). Secondly, electronic detection of package entry (by incorporating a microchip in the container or registering and time-stamping removal of a single dose: the latest systems allow for real-time transfer of information on dosing and timing to the researcher). Finally, ingestible smart sensors, embedded in pharmaceuticals, which send a time-stamped signal (activated by gastric acid) to a patch worn on the patient’s skin (known as “raisin technology”). Additional research is required, to determine the accuracy, usability, and acceptability of smart technology before their use in drug trials can be recommended.

The use of electronic monitoring devices could be considered, following positive experiences in solid organ transplantation: a multicenter RCT employed such technology successfully in 219 participants to compare medication adherence (primary endpoint) between modified-release tacrolimus once-daily and twice-daily regimens (22). The feasibility of such monitoring in kidney transplant populations could be justified, relative to the drug-development and overall costs of RCTs; return on investment could be substantial, given that it is the only method available to visualize daily drug-intake patterns. However, self-reporting of medication adherence should be embedded in all clinical trials, irrespective whether such PROMs can be combined with smart technology measures.

## PROMs for Symptoms and Side Effects

Instruments to measure the impact of symptoms and side effects should be selected based on similar criteria relating to reliability, validity, and responsiveness to change as those proposed by COSMIN (57). The instrument chosen should be determined by the RCT aims and the type of intervention. For example, an intervention that aims to alleviate gastrointestinal side effects—either through additional medication or substitution of immunosuppressants or dose adjustments—might select the GSRS and the Gastrointestinal Quality of Life Index as being relevant validated instruments (54). Similarly, instruments assessing anxiety, depression, or mood swings may be required to address the intervention aim.

Instruments can also report frequency and severity of side-effect profiles associated with immunosuppression. A systematic review applying the COSMIN checklist to appraise the psychometric quality of PROMs used in patients with kidney disease deemed the ESRD-SCLTM to be the most suitable measure for use in research and clinical practice, as it had strong evidence for internal consistency, and moderate evidence for test/retest reliability and structural and construct validity (10). However, the authors of this review also noted that no instrument had evidence supporting all measurement properties.

## PRO Reporting

We recommend that PRO reporting in study protocols should follow the international, consensus-based SPIRIT-PRO extension guidelines, with CONSORT-PRO used for reporting RCT results (4).

## Conclusions

• The use of PROMs in RCTs enables assessment of the patient’s perspective of their own health:
  ○ PROM selection requires consideration of the appropriateness, reliability, validity, acceptability, and feasibility of use.
  ○ Transparent reporting on the use and results of PROMs, using established frameworks, is required.
• The PROs life participation, medication adherence, and symptoms and side effects are suitable secondary endpoints in interventional studies.
  ○ These PROs are relevant and important in the context of kidney transplantation.
• Electronic monitoring to document adherence in RCTs is advised.
  ○ If this is not feasible, self-report measures such as the BAASIS might be considered, bearing in mind that self-reporting data has limited reliability and does not capture day-to-day patterns of medication intake or regularity of intake.
• SPIRIT-PRO should be used for reporting study protocols, and CONSORT-PRO for reporting RCT results.
• Physical, emotional, and cognitive functioning, mental health, and health-related quality of life are relevant PRO domains to be considered in trials in kidney transplantation.

### Scientific Advice from the Committee for Medicinal Products for Human Use of the European Medicines Agency Regarding These Conclusions

• CHMP acknowledged that PRO measures are important to capture the patient’s perception; nevertheless, assessment of PRO data is difficult due to the nature of such data.
  ○ The benefit/risk assessment of clinical trials addresses many of the issues participants express as being important, which include achieving long-term organ function with fewer comorbidities or adverse events.
• Among the most frequently cited instruments to address generic health status are the SF-36, the Sickness Impact Profile, and the WHO-QOL.
  ○ ESOT suggests how to establish a “core outcome set” in clinical trials of transplantation that do not incorporate instruments frequently used to address generic health status. These core outcomes should:
    – Capture recipients’ goals to fulfill their roles and re-establish a normal lifestyle
    – Include the diverse activities of “life” as defined by recipients
    – Capture life changes caused by complications and side effects of treatment
    – Be validated and feasible to implement (14).
• CHMP supported the inclusion of HRQoL measures and agreed that a validated PRO tool could be important.
  ○ PROs are often included as secondary measures of efficacy in clinical trials.
  ○ Use of a PRO as primary endpoint would require predefining a clinically meaningful improvement as measured by the PRO and powering of the study to this difference; the CHMP was not aware of a consensus defining such difference.
  ○ There is a burden in participating in clinical trial for each patient, generally higher than in normal clinical practice.
• The CHMP agreed that the selection of PROs requires consideration of the appropriateness, reliability, validity, acceptability, and feasibility of use, without causing excess burden to the study participant.
  ○ The proposed SPIRIT-PRO extension guideline and use of the CONSORT-PRO for reporting the results of randomized trials (4) are acceptable; guidelines on reporting files from clinical trials are provided in GCP guideline EMA/INS/GCP/856758/2018.
• The CHMP agreed that medication adherence should be measured by reliable methods in clinical trials, considering ICH E9 (R1) addendum on estimands and sensitivity analyses should be performed.
  ○ The count of returned tablets is not deemed a fully reliable measure of adherence, but it is an important tool used in clinical trials.
  ○ Medication adherence is known to be better during clinical trials and to decrease over the course of regular treatment, especially if the treatment is lifelong; therefore, medication adherence is an important PRO to evaluate.
    – However, the assessment of medication adherence in a clinical trial could prove to be difficult to extrapolate to real life.
  ○ Measures involving electronic medication packaging and different smart technologies are gaining traction for measuring adherence and could provide more robust information than questionnaires, interviews, and scales.
